# The Cog-4 Subset of the National Institutes of Health Stroke Scale as a Measure of Cognition: Relationship with Baseline Factors and Functional Outcome after Stroke Using Data from the Virtual International Stroke Trials Archive

**DOI:** 10.1155/2013/562506

**Published:** 2013-03-26

**Authors:** Sandeep Ankolekar, Cheryl Renton, Nikola Sprigg, Philip M. W. Bath

**Affiliations:** ^1^Stroke Trials Unit, Division of Stroke, University of Nottingham, City Hospital Campus, Nottingham NG5 1PB, UK; ^2^Stroke Service, City Hospital, Nottingham University Hospitals NHS Trust, Nottingham NG5 1PB, UK

## Abstract

*Background*. Assessing poststroke cognitive impairment is complex. A subscale of the NIHSS, the Cog-4, has been proposed as a quick test of “cognitive impairment.” but a study of its properties in a larger dataset is lacking. *Methods*. Data from 9,147 patients with acute stroke from the VISTA archive was used to generate Cog-4 scores. The statistical properties of Cog-4, its relationship with baseline clinical characteristics, and other functional outcome measures at day 90 were assessed. *Results*. Mean age of patients was 69.2 years and 45.8%, were females. Day-90 Cog-4 was highly positively skewed (skewness 0.926). Patients with left hemispheric stroke had higher day-90 Cog-4 score (*P* < 0.001). Age, stroke severity, and previous stroke were significant predictors of Cog-4. Cog-4 was significantly correlated with dependency (modified Rankin Scale, *r*_*s*_ = 0.512), and disability (Barthel Index, *r*_*s*_ = −0.493). *Conclusions*. The Cog-4 scale at day 90 cannot be considered a useful test of cognition since it only superficially measures cognition. It is heavily dependent on the side of stroke, is inevitably associated with functional outcome (being a subset of the NIHSS), and suffers from a profound “floor” effect. Specific and validated measures are more appropriate for the assessment of poststroke cognition than Cog-4.

## 1. Introduction

Poststroke cognitive impairment (PSCI) is an important but poorly studied consequence of stroke and is a significant risk factor for developing frank dementia [1, 2]. PSCI diagnosed in the first few months after stroke may progress to dementia, remain stable, or improve over the following months to years [3, 4]. It is important to understand factors that are responsible for the development of PSCI, and study the impact of PSCI on other functional outcomes to develop preventative and management strategies. 

However, research on PSCI has been hindered, partly by the relative lack of relevant measures of cognition and standardised diagnostic criteria to identify this condition, and partly by the lack of use of these in acute stroke and secondary prevention trials [5]. It is well established that the neurocognitive profile of PSCI, poststroke dementia, and vascular dementia differs from Alzheimer's disease, the most common type of dementia [6–8], but their frequent coexistence can cause diagnostic challenges. Vascular dementia typically damages executive function and yet standard cognitive screening tests such as the Mini-Mental State Examination (MMSE) lack a significant measure of executive component [9, 10]. A number of newer cognitive screening tests, including the Montreal Cognitive Assessment [11] and Addenbrooke's Cognitive Examination [12], address some of these deficits. Indeed, the “Vascular Cognitive Impairment Harmonisation Standards” committee recommend a battery of tests that cover all the important cognitive domains, including executive function [13].

The National Institutes of Health Stroke Scale (NIHSS) is a standardised clinical scale, that is well established as a quantitative measure of stroke severity and impairment [14]. Recently, a subset of the NIHSS, Cog-4 (Table 1) [15], corresponding to questions 1b (orientation), 1c (response to commands), 9 (language), and 11 (extinction or inattention), has been proposed as a quick test of cognitive impairment in poststroke patients [15]. The present study further characterises the Cog-4 as a potential marker of cognitive impairment after stroke and assesses its relationship with baseline predictors of cognition and functional outcome measures such as disability and dependency. The study used data from the VISTA archive of trial data [16].

## 2. Methods

### 2.1. Virtual International Stroke Trials Archive (VISTA)

VISTA is an international academic collaboration that has been established to promote excellence in stroke care and trial design [16]. It contains data on more than 28,000 participants from stroke studies in acute stroke, intracerebral haemorrhage, rehabilitation, secondary prevention, and observational stroke studies. The acute stoke trials archive includes both ischaemic and haemorrhagic strokes. VISTA does not share information on the precise trials acting as the source of data for analyses (http://www.vista.gla.ac.uk/).

### 2.2. Patients

Patients from the VISTA acute stroke trials archive were included if they had data measured at day 90 on the NIHSS (providing individual components was available), modified Rankin Scale (mRS) [17], and Barthel Index (BI) [17]. Additional information was used including baseline demographics, risk factors and index stroke information. 

### 2.3. Cog-4

The Cog-4 [15] comprises four items from the NIHSS (Table 1): level of consciousness (1b), which measures orientation; level of consciousness (1c) assessing the ability to follow commands; language (9); and inattention (11). These components individually score 0–2 or 0–3 points giving the Cog-4 scale, a score that ranges from 0 to 9 points. Of importance is that the Cog-4 was originally reported in a population of patients 1.5 years after stroke [15].

### 2.4. Assessments

Point estimates and the distribution of the Cog-4 were calculated. The effect of lesion side on the Cog-4 was then assessed making the assumption that most patients will have been right handed and many of the minority who were left handed would also have been cortically left dominant. The absence of any formal cognition data in the acute VISTA dataset (reflecting that, historically, acute stroke trials have not measured this parameter at outcome) precluded any comparison of Cog-4 with the MMSE or other cognitive assessment tools.

Univariate and multiple variable relationships between Cog-4 and potential baseline predictors of cognition (age, sex, stroke risk factors, stroke side, type of stroke (ischaemic, haemorrhagic) and stroke severity) were studied, as was the relationship of the Cog-4 with mRS and BI, all measured at day 90. The relevance of including death in the Cog-4 scale was also studied where death was assigned a score of 10, that is, one more than the maximum score of 9, as is done with the mRS (death = 6) and BI (death = −5).

The effect of treatment with alteplase on day-90 Cog-4 was assessed with adjustment for age, sex, and baseline NIHSS in a separate analysis performed only in patients with ischaemic stroke.

### 2.5. Statistical Methods

Data are described as number (%), median [interquartile range], mean (standard deviation), skewness, and kurtosis. Comparisons between groups were assessed using the Chi Square Test (binary data) and Mann-Whitney *U* test (ordinal data). Univariate relationships were assessed using Spearman's rank correlation coefficient for continuous variables and Somer's D for categorical variables. Multiple variable analysis was performed using backward elimination, ordinal logistic regression with *P* > 0.10 as the criteria for exclusion. Statistical analysis was performed using the Statistical Package for Social Sciences, version 19 (SPSS-19) for Mac. Significance was set at *P* < 0.05.

## 3. Results

From the original VISTA dataset of 11,648 patients, 2,501 patients were excluded due to missing data, 1,850 patients for absent data at day 90, and 651 patients for missing data at baseline. The analyses included 9,147 patients with acute ischaemic (90.8%) or haemorrhagic strokes (9.2%) (Table 2), with mean age 69.2, being a female 45% and mean NIHSS 13. Data on the Oxfordshire Community Stroke Project (OCSP) classification [18] was available for 1,703 participants; lacunar (13.5%) and posterior circulation (3.6%) strokes were underrepresented in the sample. Strokes occurred equally between the left and right hemispheres (49 versus 51%). 1,902 (20.8%) patients died by day 90, so NIHSS scores were available for 7,245 (79.2%).

### 3.1. Cog-4 at Day 90

**Figure 1:**
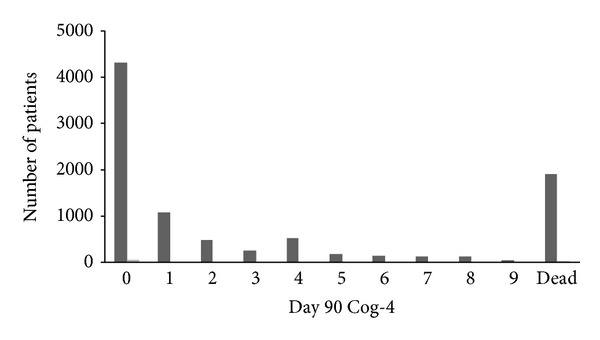
Distribution of Cog-4 at day 90.

The median [interquartile range (IQR)] Cog-4 on day 90 was 0 [0–2]; if death was included, the median was 1 [0–6]. Of all participants 47.2% had a normal Cog-4 score of 0 at day 90 (as compared to 23.9% at baseline) and the distribution was skewed to the right (skewness = 0.936, kurtosis −0.846, Figure 1). Participants with a left hemispheric stroke had a higher median score [IQR] as compared to those with right hemispheric stroke; Cog-4, 1 [0–7] versus 0 [0–4] (Mann-Whitney *U*, *P* < 0.001), and statistically significant differences were present for each of the individual items of the score (Table 3). The percentage of abnormal responses for each item decreased between baseline and day 90.

### 3.2. Baseline Predictors of Cog-4 at Day 90

Increasing age, stroke severity (NIHSS), female sex, and a history of atrial fibrillation, previous stroke or hypertension, were significantly associated with a higher day 90 Cog-4 score on univariate analysis (*r*_*s*_ > 0.1 for age, atrial fibrillation and stroke severity) (Table 4). Other factors such as diabetes mellitus, history of smoking and haemorrhagic stroke (*r*_*s*_ = 0.14, Table 4) were also significant when Cog-4 was extended to include death (score 10). Smoking was not included in the multiple variable analysis as data were missing for 5,570 patients. On multiple variable analysis (ordinal logistic regression), increasing age or stroke severity and a history of previous stroke continued to remain significant (*P* < 0.001, Table 4) in both regression models with Cog-4 including or excluding death. Additionally, a history of atrial fibrillation (Cog-4 with death, *P* = 0.085; Cog-4 without death, *P* = 0.063) or diabetes (Cog-4 with death, *P* = 0.052) approached significance. 

### 3.3. Relation between Cognition and Other Functional Outcomes

Higher Cog-4 scores were associated with increased dependency (mRS) and disability (BI) (Table 4). Associations with functional outcomes were numerically larger when death was included in the cognition scale (Table 4).

### 3.4. Effect of Treatment with Alteplase on Cog-4

In patients with ischemic stroke, Cog-4 score at day 90 was lower in patients treated with alteplase as compared with those not receiving alteplase, 0 [0–4] versus 1 [0–7] (*P* < 0.001), and remained significant following adjustment for age, sex and NIHSS at baseline.

## 4. Discussion

The aim of the present study was to assess the construct and the clinical implications of the statistical properties of Cog-4, study its relationship with baseline prognostic factors and functional outcomes of dependency and disability, and assess the effect of rt-PA on it. Clinimetrically, Cog-4 is highly positively skewed (mode 0, median 1, and mean 3.05) with more than 40% of patients having a 0 score at day 90. Inclusion of death in the score (as is done with other functional measures including mRS and BI) creates a bimodal distribution (modes 0 and 10) with a profound floor and ceiling profile.

Cog-4 was significantly associated, in multiple variable analyses, with classical baseline prognostic factors, in particular increasing age, stroke severity, and history of previous stroke. The association of cognition with age [19–24], stroke severity [20, 25], and previous stroke [24, 26, 27] has been shown in previous studies, and is plausible biologically since large and previous strokes lead to more neuronal loss and destruction of cognitive circuits, especially in an aging brain. However, since Cog-4 is a derivative of NIHSS, it is inevitably associated with both age and severity like the NIHSS, so these associations do not imply that Cog-4 is a useful measure of cognition. Further, age and severity are the two most powerful predictors of functional outcome [28], so again the relationship between Cog-4 and dependency and disability is inevitable, and not because Cog-4 is measuring cognition. 

As a result, the use of Cog-4 as a cognitive measure following stroke, as proposed by Cumming et al., has to be questioned. First, it contains a poor assessment of executive function, a requisite for any test that aims to assess PSCI. The original paper proposing Cog-4 suggested that question 1c of the NIHSS assesses executive function [15] although it is probably more appropriate as a marker of language comprehension. Undoubtedly, attention and normal frontal lobe function are necessary for answering one- and two-stage commands appropriately, and some severely aphasic patients with left perisylvian lesions may still maintain the ability to correctly answer one- and two-stage commands, particularly involving axial musculature [29]. Nevertheless, this item is more often found to be abnormal with left-sided lesions causing reduced comprehension as reported earlier [30] and is seen in the present study, both at baseline and day 90.

Second, Cog-4 not only inherits but magnifies the shortcomings of the NIHSS [30] in having more items assessing left-sided function compared to these assessing right-sided. Three of the four items in Cog-4 were more likely to be abnormal with left-sided lesions as compared with functional right indentation. Consequently, Cog-4 will lack sensitivity in patients with right-sided strokes. Third, the constitution of the score is such that items 1b, 1c, and 9 assess language and item 11 assess, sensory inattention. Hence, patients with severe abnormalities in only one or two cognitive domains may be misclassified as severely impaired. Furthermore, due to the rules and conventions used in scoring the NIHSS, patients with severe dysphasia will be scored maximally on the LOC questions.The lack of even a simple measure of memory, a domain perhaps less affected in PSCI but certainly important in the long term for independent living and development of dementia, also makes the test less than adequate.

Fourth, the test suffers from significant floor effects; excluding patients who died by day 90, only 20.3% had a score more than 1 on the Cog-4 scale. The test is unlikely to be sensitive for patients with mild cognitive impairment, irrespective of the side of stroke, where the effect of any interventional strategies is most likely to be beneficial. That the test was comparable to the MMSE in the original validation study [15], perhaps reflects the insensitivity of the MMSE as a test for PSCI.

Fifth, use of the Cog-4 as an outcome measure may be flawed because of its derivation. Inevitably, a subset of the NIHSS is going to be strongly associated with the NIHSS itself, and because the baseline NIHSS is the most powerful predictor of subsequent functional outcome, then a sub-score of the NIHSS is again likely to be strongly related to other functional outcome measures.

The Cog-4, as published, ignores people who die, a sizable proportion after acute stroke and 21% in the present study. But patients who die in trials cannot be ignored, not least because a treatment that could “kill or cure” would never have the hazard detected if fatal outcomes were ignored. As a result, the mRS and BI have been expanded to include those who die (mRS = 6, BI = −5) although the cause of death is ignored so the same score is used whether the patient dies of their index event, sepsis, myocardial infarction, pulmonary embolism, or any other cause. As a result, other functional outcome measures used in acute stroke should also be expanded to include death. In the present study, we assigned a greater than maximal score to death, that is, Cog-4 = 10 points. Although this means that the Cog-4 has a bimodal distribution (with both floor and ceiling effects), the adjustment increased the number of baseline factors (addition of diabetes) that were predictive of the final Cog-4 score and gave numerically higher Spearman correlation coefficients with other functional outcome measures.

The present study has several limitations. First, we were unable to directly validate the Cog-4 with other detailed cognitive assessments at day 90 since acute stroke trials have, until recently, not collected such data (hence their absence in the acute VISTA archive). Such an exercise would provide evidence, or lack of it, for the external validity and reliability of Cog-4. Second, there was no evaluation of prestroke cognitive impairment and it is difficult to say if factors other than the index stroke contributed to the Cog-4 score at day 90. Third, while this is perhaps the largest single study to assess predictors of potential PSCI in the subacute stroke period, the results should be interpreted with caution, given the use of the Cog-4 as a putative test of cognition. Nevertheless, the present study is based on a large sample size and incorporates high fidelity data from a number of randomised controlled trials.

In summary, the Cog-4 at day 90 has a highly positively skewed distribution with more than 40% of patients scoring 0. Since it is biased towards language performance, its score is highly dependent on the side of the stroke, that is, patients with left-sided stroke have higher scores. Cog-4 at day 90 is related to age, baseline severity, history of previous stroke, and functional outcome (mRS and BI). We are not aware of any studies that have used the Cog-4 as a cognitive measure, but its serious shortcomings suggest that use of existing multidimensional measures of cognition is likely to be more appropriate.

## Figures and Tables

**Table 1:** Items of the NIHSS selected to form the Cog-4 cognitive scale with range 0–9.

	Cog-4	Score
(1b)	Level of consciousness- questions [month and age]	
	Answers both correctly	0
	Answers one question correctly	1
	Answers neither questions correctly	2
(1c)	Level of consciousness commands (open and close eyes; grip and release hand)	
	Performs both tasks correctly	0
	Performs one task correctly	1
	Performs neither task correctly	2
(9)	Best language	
	No aphasia	0
	Mild to moderate aphasia	1
	Severe aphasia	2
	Mute and global aphasia	3
(11)	Extinction and inattention	
	No inattention	0
	Mild inattention	1
	Severe inattention	2

**Table 2:** Baseline characteristics; mean (SD) or number (%).

Variables	
Total number of participants	9,147
Age (years)	69.2 (12.5)
Sex, male (%)	4957 (54.2)
Risk factors (%)	
Atrial fibrillation	24.7
Diabetes	22.0
Hypertension	68.5
Previous MI	14.0
Previous stroke/TIA	28.3
Left hemispheric strokes (%)	49.0
NIHSS score (/42)	13.1 (5.8)
Cog-4	1 [0–6]
Stroke type (%)	
Ischaemic stroke	90.8
Primary intracerebral haemorrhage	9.2
Stroke syndrome (*n* = 1703) (%)	
Total anterior circulation	42.3
Partial anterior circulation	40.6
Lacunar	13.5
Posterior circulation	3.6

**Table 3:** Comparison of abnormal responses (score > 0) on Cog-4 between right and left hemisphere strokes (values in percentage).

Variable	Baseline			Day 90		
			*P* value			*P* value^†^
	Left	Right		Left	Right	
Orientation	66.3	20.3	<0.001	33.9	16.3	<0.001
Commands	42.7	8.3	<0.001	19.4	8.9	<0.001
Language	76.3	14.0	<0.001	47.4	12.8	<0.001
Extinction	36.8	62.9	<0.001	20.4	28.5	<0.001

Total	83.5	69.0	<0.001	59.6	46.3	<0.001

^†^Chi-square test.

**Table 4:** Univariate relationships between Cog-4 and baseline variables and day 90 functional outcome. Association by Spearman's rank correlation for continuous variables and Somer's *D* for categorical variables. Patients who died were assigned a score of 10 on the Cog-4 in the analysis with death included.

	Without	Death	With	Death
	*r*_*s*_	2*P*	*r*_*s*_	2*P*
Baseline				
Age	0.139	<0.001^†^	0.254	<0.001^†^
Sex, female	0.035	0.004	0.032	0.005
Atrial fibrillation	0.133	<0.001	0.210	<0.001
Diabetes mellitus	0.011	0.447	0.053	<0.001
Hypertension	0.030	0.019	0.045	<0.001
Previous stroke or TIA	0.034	0.015^†^	0.075	<0.001^†^
History of smoking	0.056	0.006	−0.149	<0.001
Stroke severity (NIHSS)	0.503	<0.001^†^	0.505	<0.001^†^
Stroke type (IS, PICH)	0.044	0.288	0.141	<0.001
Left hemisphere stroke	0.206	<0.001	0.017	<0.001
Functional outcomes				
Modified Rankin Scale	0.512	<0.001	0.772	<0.001
Barthel Index	−0.493	<0.001	−0.766	<0.001

^†^Significant on multiple variable analysis.

IS: ischaemic stroke; PICH: primary intracerebral haemorrhage.

2*P*: 2-sided *P* value.

*r*_*s*_: correlation coefficient.

## References

[1] Srikanth VK, Quinn SJ, Donnan GA, Saling MM, Thrift AG (2006). Long-term cognitive transitions, rates of cognitive change, and predictors of incident dementia in a population-based first-ever stroke cohort. *Stroke*.

[2] Serrano S, Domingo J, Rodríguez-Garcia E, Castro MD, Del Ser T (2007). Frequency of cognitive impairment without dementia in patients with stroke: a two-year follow-up study. *Stroke*.

[3] Ballard C, Rowan E, Stephens S, Kalaria R, Kenny RA (2003). Prospective follow-up study between 3 and 15 months after stroke: improvements and decline in cognitive function among dementia-free stroke survivors >75 years of age. *Stroke*.

[4] Rasquin SMC, Lodder J, Verhey FRJ (2005). Predictors of reversible mild cognitive impairment after stroke: a 2-year follow-up study. *Journal of the Neurological Sciences*.

[5] Ankolekar S, Geeganage C, Anderton P, Hogg C, Bath PMW (2010). Clinical trials for preventing post stroke cognitive impairment. *Journal of the Neurological Sciences*.

[6] Looi JCL, Sachdev PS (1999). Differentiation of vascular dementia from AD on neuropsychological tests. *Neurology*.

[7] Desmond DW (1996). Vascular dementia: a construct in evolution. *Cerebrovascular and Brain Metabolism Reviews*.

[8] Garrett KD, Browndyke JN, Whelihan W (2004). The neuropsychological profile of vascular cognitive impairment—no dementia: comparisons to patients at risk for cerebrovascular disease and vascular dementia. *Archives of Clinical Neuropsychology*.

[9] Desmond DW, Moroney JT, Bagiella E, Sano M, Stern Y (1998). Dementia as a predictor of adverse outcomes following stroke: an evaluation of diagnostic methods. *Stroke*.

[10] Pendlebury ST, Cuthbertson FC, Welch SJV, Mehta Z, Rothwell PM (2010). Underestimation of cognitive impairment by mini-mental state examination versus the montreal cognitive assessment in patients with transient ischemic attack and stroke: a population-based study. *Stroke*.

[11] Nasreddine ZS, Phillips NA, Bédirian V (2005). The Montreal Cognitive Assessment, MoCA: a brief screening tool for mild cognitive impairment. *Journal of the American Geriatrics Society*.

[12] Mioshi E, Dawson K, Mitchell J, Arnold R, Hodges JR (2006). The Addenbrooke’s Cognitive Examination revised (ACE-R): a brief cognitive test battery for dementia screening. *International Journal of Geriatric Psychiatry*.

[13] Hachinski V, Iadecola C, Petersen RC (2006). National Institute of neurological disorders and stroke-Canadian stroke network vascular cognitive impairment harmonization standards. *Stroke*.

[14] Brott T, Adams HP, Olinger CP (1989). Measurements of acute cerebral infarction: a clinical examination scale. *Stroke*.

[15] Cumming TB, Blomstrand C, Bernhardt J, Linden T (2010). The NIH stroke scale can establish cognitive function after stroke. *Cerebrovascular Diseases*.

[16] Ali M, Bath PMW, Curram J (2007). The virtual international stroke trials archive. *Stroke*.

[17] Sulter G, Steen C, De Keyser J (1999). Use of the Barthel Index and Modified Rankin Scale in acute stroke trials. *Stroke*.

[18] Bamford J, Sandercock P, Dennis M, Burn J, Warlow C (1991). Classification and natural history of clinical identifiable subtypes of cerebral infarction. *Lancet*.

[19] Censori B, Manara O, Agostinis C (1996). Dementia after first stroke. *Stroke*.

[20] Barba R, Martínez-Espinosa S, Rodríguez-García E, Pondal M, Vivancos J, Del Ser T (2000). Poststroke dementia: clinical features and risk factors. *Stroke*.

[21] Tamam B, Taşdemir N, Tamam Y (2008). The prevalence of dementia three months after stroke and its risk factors. *Turkish Journal of Psychiatry*.

[22] Rasquin SMC, Verhey FRJ, Van Oostenbrugge RJ, Lousberg R, Lodder J (2004). Demographic and CT scan features related to cognitive impairment in the first year after stroke. *Journal of Neurology, Neurosurgery and Psychiatry*.

[23] Zhou DHD, Wang JYJ, Li J, Deng J, Gao C, Chen M (2004). Study on frequency and predictors of dementia after ischemic stroke: the chongqing stroke study. *Journal of Neurology*.

[24] Lin JH, Lin RT, Tai CT, Hsieh CL, Hsiao SF, Liu CK (2003). Prediction of poststroke dementia. *Neurology*.

[25] Jaillard A, Grand S, Le Bas JF, Hommel M (2010). Predicting cognitive dysfunctioning in nondemented patients early after stroke. *Cerebrovascular Diseases*.

[26] Hénon H, Durieu I, Guerouaou D, Lebert F, Pasquier F, Leys D (2001). Poststroke dementia: incidence and relationship to prestroke cognitive decline. *Neurology*.

[27] Pohjasvaara T, Erkinjuntti T, Ylikoski R, Hietanen M, Vataja R, Kaste M (1998). Clinical determinants of poststroke dementia. *Stroke*.

[28] Counsell C (1998). *The prediction of outcome in patients with acute stroke [dissertation]*.

[29] Kasper DL (2005). Aphasia, memory loss, and other focal cerebral disorders. *Harrison'S Principles of Internal Medicine*.

[30] Woo D, Broderick JP, Kothari RU (1999). Does the National Institutes of Health Stroke Scale favor left hemisphere strokes?. *Stroke*.

